# Supplementary material for: Review of the application of the open-source software CilOCT for semi-automatic segmentation and analysis of the ciliary muscle in OCT images
Source: PLoS One. 2020 Jun 9;15(6):e0234330. doi: 10.1371/journal.pone.0234330 (PMC7282635; doi:10.1371/journal.pone.0234330)
Supplement: Attachment — Submitted filename: review CilOCT.docx [file pone.0234330.s004.docx]

Thank you for allowing me to review this interesting paper.

Research article-is the very suitable for this article.

Open-Source is the most talked issue in programing in the last years It’s very good to see it in our field.

Remarks

abstract

1. I think you need to elaborate more about the “batch processing”- Most of the readers don’t know enough about it.

Intro

2. You are talking about different refractive Indices please elaborate more including the numbers you are talking about.
3. You’re explaining how you took the images- Its not very clear- please add a photo how you’ve taken it. Moreover- you’re siting [16] your own article.

Method

4. When you are talking about figure 2e and 2f is not very clear. It needs to be more elaborated.
5. The colors in 2b are not sharp enough- very difficult to see it and understand.
6. In line 126 you are talking about “distortion correction”- it is not clear enough since you’ve already talked about the ciliary muscle in 2d.

Discussion

7. You are talking about 6 OCT images in 15 Volunteers – why is the discrepancy?

I think it is a very interesting research article with a very updated topic. Though with a small number of subjects.
