# Supplementary material for: Review of the application of the open-source software CilOCT for semi-automatic segmentation and analysis of the ciliary muscle in OCT images
Source: PLoS One. 2020 Jun 9;15(6):e0234330. doi: 10.1371/journal.pone.0234330 (PMC7282635; doi:10.1371/journal.pone.0234330)
Supplement: Attachment — Submitted filename: Response to Reviewers.docx [file pone.0234330.s005.docx]

# Reviewer #1

Thank you for allowing me to review this interesting paper.

Research article-is the very suitable for this article.
Open-Source is the most talked issue in programing in the last years It’s very good to see it in our field.

We thank the Reviewer for the appraisal and for the valuable comments on our manuscript. We fully agree with the statement that open-source is important, especially but not only in biomedical research, as making software and algorithms publicly available will foster reproducibility in science.

## abstract

1. I think you need to elaborate more about the “batch processing”- Most of the readers don’t know enough about it.

We changed the description of the batch processing feature in the Material and methods section as follows:

The batch-processing feature allows for the ***~~processing~~*** ***analysis*** of large ~~datasets~~ numbers of images ***~~by~~*** based on ~~using~~previously created and stored XML segmentation files ***and to subsequently export the anatomical ciliary muscle landmarks, determined parameters, and CMT profiles as tabulated text files.***

Additionally, we extended the supplementary movie S1 by a demonstration of the application of the batch processing.

**Intro**
2. You are talking about different refractive Indices please elaborate more including the numbers you are talking about.

We added the values of the refractive indices as well as a reference to the source we used. Furthermore, we adjusted the text as follows:

Before the determination of ciliary muscle biometric parameters, the image distortion ~~due~~***resulting from the refraction of the infrared light beam on boundaries*** ~~to the~~ ***with*** different refractive ***indices is corrected to enable the calculation of the true layer thickness. Image distortion correction is performed and applied to the segmentation using ray-tracing based on Snell’s Law and the refractive indices of the respective layers*** ~~of the tissue layers~~***(sclera: 1.41, ciliary muscle: 1.38, aqueous fluid: 1.34 [19])***~~can be corrected and applied to the segmentation using ray tracing and Snell’s Law~~.

3. You’re explaining how you took the images- Its not very clear- please add a photo how you’ve taken it. Moreover- you’re siting [16] your own article.

We moved the description of how the images of the ciliary muscle were taken to the Material and methods section. Additionally, we give more information about the process and explicitly point to our previous article, which provides a detailed description as well as a sketch of the experimental setup. Since the focus of the manuscript is the description of the open-source software CilOCT we developed and would like to provide to other researchers, and since the process and the setup have already been described in previous articles, we think adding a picture is not necessary. However, citing our article is inevitable.

The updated text:

***In our previous studies, a Zeiss Visante^TM^ AS-OCT (Carl Zeiss Meditec AG, Jena, Germany; enhanced high-resolution corneal mode, 512 A-scans, scanning size: 10 mm x 3 mm, wavelength 1310 nm) was used. To image the ciliary muscle, subjects were positioned in front of the OCT device with their head placed in the combined head and chin rest. After adjusting the imaged area using pupil center alignment, the subjects were instructed to shift their gaze about 40° to an external target without moving their head but keeping it parallel to the instrument. This allowed imaging the entire ciliary muscle as shown in Fig 1. A detailed description of the process including a sketch of the experimental setup can be found in [20].***

## Method

4. When you are talking about figure 2e and 2f is not very clear. It needs to be more elaborated.

We rephrased the corresponding paragraph to clarify the distortion correction based on Snell’s Law as follows:

In addition to the ciliary muscle borders, the scleral-air boundary, as well as the borders of the anterior chamber are segmented by a simple 8-neighbors flood-fill algorithm using a manually placed starting point (purple landmark in Fig 2b, turquoise area in Fig 2c, orange and yellow borders in Fig 2d). These borders are required for the following distortion correction ***using Snell’s Law and*** ~~Fig 2e~~***the refractive indices of the different tissues (infrared light rays depicted as yellow lines perpendicular to the upper border of the image and as red/orange lines after refraction at the different tissue borders in Fig 2e).*** ~~In a similar way~~Similarly, a possibly worn contact lens can be segmented (***see S1 movie for an example~~not shown~~***). The thresholds of the flood-fill algorithm can be manually adjusted for the different regions. **Fig 2f depicts the determined biometric parameters scleral spur, ciliary muscle apex, perpendicular axis, and ciliary muscle area after distortion correction.**

5. The colors in 2b are not sharp enough- very difficult to see it and understand.

We enlarged the size of the guiding landmarks for better visibility.

6. In line 126 you are talking about “distortion correction”- it is not clear enough since you’ve already talked about the ciliary muscle in 2d.

As described in the section “Segmentation of the ciliary muscle using CilOCT” (formerly “Illustrative Example”), a distortion correction using the refractive indices of the different tissue layers is performed after the semi-automatic segmentation and before the calculation of the biometric parameters and the ciliary muscle thickness profile. The legend of Fig. 2 was updated to better explain the process.

## Discussion

7. You are talking about 6 OCT images in 15 Volunteers – why is the discrepancy?

Thank you for pointing out this misleading phrase. Six OCT were taken in each volunteer for near and far accommodation, respectively. We changed the text as follows:

Six OCT images of the right temporal ciliary muscle ***were taken in each*** of ***the*** volunteers ***~~were imaged~~*** during both, near and far accommodation.

I think it is a very interesting research article with a very updated topic. Though with a small number of subjects.

Thank you very much for your positive feedback. We agree that the first study we referred to includes only a small number of subject. However, the main purpose of the study was to evaluate the reliability of our software and, therefore, a large number of OCT images per subject was assessed. The second study comprises 38 (18 emmetropes, 20 myopes) and the third study 35 (17 emmetropes, 18 myopes) subjects. We now explicitly added the number of subjects of the second and third study in the Discussion section.

# Reviewer #2: editor comments

## General

1 was intrigue by the title of this study – to evaluate the ciliary muscles – indeed a great topic as accommodation presbyopia and myopia our daily life.
2 The ability to standardized reports of imaging is of benefit to all researchers as it create a common language for definition and classification

We thank the Editor for appreciating our work and for the valuable comments and suggestions for improving the manuscript. We are delighted to see that the Editor shares our opinion about open-source software in research. We strongly believe that providing the software used for analyzing data, in addition to the data itself, helps in ensuring reproducible research.

## cover letter

1 I understand you offer the code for the program. Can you offer the software itself for those who do not have coding experience? An online tool will be great

Download links for both, source code and a ready-to-use pre-built version of CilOCT are given in the manuscript. Unfortunately, due to the complexity of the software, it cannot be implemented as an online tool. However, since CilOCT is developed in Java, it can be used on any operating system for which a Java Runtime Environment is available.

## Intro

1 some font and formatting could be improved- please go over and make sure all is correct= for example reference 15 is not in the same font

Thank you. We double-checked the manuscript style templates as well as all fonts.

2 FIG 1- please refer the reader to the figure from the text.

The figure is now referred to in the preceding paragraph “Imaging of the ciliary muscle using OCT” in the Material and methods section.

3 “CilOCT supports the import of images of the ciliary muscle either from standard image format 54 (BMP, JPEG, PNG) or from raw Zeiss DICOM files [15]. In the case of an import from raw Zeiss 55 DICOM format, the images are automatically rotated and resized [14].”

We removed this paragraph since it is repeated in Material and methods / Software functionalities.

and
“To image the ciliary muscle subjects were positioned in front of an anterior segment-OCT (VisanteTM 62 AS-OCT, Carl Zeiss Meditec AG, Jena, Germany; enhanced high-resolution corneal 63 mode, 512 A-scans, scanning size: 10 mm x 3 mm, wavelength 1310 nm) with their head placed 64 in a combined head and chin rest [16]. Since the infrared beam can only penetrate the sclera but 4 65 not the highly pigmented iris [17], the subjects were instructed to shift their gaze about 40° to an 66 external target while keeping their head parallel to the instrument. This allowed imaging the entire 67 ciliary muscle as shown in Fig 1 [16]. Images and results reported were collected in previously 68 published studies [16,18,19]. The studies followed the tenets of the declaration of Helsinki and 69 were approved by the Institutional Review Board of the medical faculty of the University of 70 Tuebingen (376/2017BO2)”
should move to the methods

We moved the respective paragraph to the Material and methods section.

4 The intro lacking the purpose of this study: please state clearly that this is a review study aiming to introduce the software- no new data is given her- this is an overview report
you could add that to the title that it is a review

We changed the title accordingly to “***Review of the application of the open-source software CilOCT for semi-automatic segmentation and analysis of the ciliary muscle in OCT images***” and added a new paragraph to underline the intention of the manuscript:

Here we present the first software for semi-automatic segmentation and standardized analysis of the ciliary muscle, CilOCT, which is free to use and fully open-source. Biometric parameters of the ciliary muscle are automatically calculated after segmentation and image distortion correction, accounting for the different refractive indices of the tissue layers. Additionally, CilOCT performs the formation of continuous ciliary muscle thickness (CMT) profiles, which allow for the comparison of the ciliary muscle morphology between different subjects or different accommodative demands. CilOCT provides an audit trail documenting all processing steps, fostering reproducibility of research. The application of CilOCT is demonstrated by summarizing the results of three previously published studies from our group.

## methods-

1 CilOCT is developed---> “was”

Thank you, corrected.

2 The user interface leverages the ribbon-paradigm to simplify and accelerate user interaction- please explain in words that average human can explain that is this ribbon? give example or photo?

The ribbon paradigm was introduced by Microsoft in Office 2017. We added the following text to explain the meaning of “ribbon”:

The user interface leverages the ribbon-paradigm***, a graphical control element consisting of a set of toolbars placed on several tabs, first introduced in Microsoft Office 2007, using the open-source library FXRibbon (Version 1.0.1, BSD license),*** to simplify and accelerate user interaction.

3 Table 1 – please refer the reader from the text- I think this could be incorporated in the text actually as the table does not add much

We removed Table 1 and incorporated its content into the text instead.

4 ccontrast can either be manually increased or decreased or adjusted by adaptive histogram equalization--->Is different contrast affect the measurements?

Changing the contrast can help to better identify the border between different layers of tissue and to place the guiding landmarks along the ciliary muscle’s boundaries. However, this does not affect the following semi-automated segmentation and has no effect on the measurements.

We modified the respective description as follows:

After importing the image, its contrast can either be manually increased or decreased or adjusted by adaptive histogram equalization [18], to enhance the edges of the ciliary muscle ***for an easier placement of guiding landmarks along the muscle’s boundaries. Changing the contrast does not affect the following automatic segmentation***.

5 Illustrative Examples – could you provide capture screen video as a tutorial? ah S1 movie Ok

A screen recording of an exemplary segmentation of the ciliary muscle in an OCT image is provided as a supplementary movie (S1 Movie). Additionally, we extended the video by a demonstration of the application of the batch processing feature.

6 is a windows version exist that a regulat user can download and install and work?

In addition to the source code, a pre-built version of CilOCT is available for download from Github. Both URLs are now given in the manuscript in the Results section.

Since CilOCT is developed in Java, it can be used with all operating systems for which a Java Runtime Environment is available. These are amongst others: Windows, macOS, and Linux/Unix.

## Discussion

1 no results sections?

There was indeed no Results section. We find it challenging to introduce an open-source software, which we want to provide for free to the research community, in the commonly used structure of research articles. However, we now changed the structure of the article to the common form and described the application of CilOCT and its availability in the Results section.

for example, from 100 scans how many were accurate? what is the false positive negative? timing of the procedure? etc.

Unfortunately, at this point we are not able to provide such information, since there is currently no “gold-standard” for ciliary muscle segmentation and analysis. Moreover, software other groups used to segment and analyze the ciliary muscle in OCT images is often proprietary and not available. We hope that the publication of this manuscript and providing CilOCT as open-source will enable the comparison of different approaches in future.

2 The intra-examiner analysis of the CMA revealed---where is this reported? how many examiners? how many test? what was the statistical test

Intra-examiner analysis was performed by comparing the results of two segmentations performed consecutively by the same examiner (SW). The details of the experiment, the analysis, the statistical tests and the results are published in the referred article. We therefore do not think that it is necessary to repeat this here.

please follow a intro- method-results-conclusion design- we are all used to it

We changed the structure of the manuscript to the common introduction – methods – results – discussion – conclusion design.

ciliary muscle thickness profiles- you should show that this is indeed an helpful parameter

We added the following paragraph to the Discussion section in order to point out the usefulness of the ciliary muscle thickness profiles:

Furthermore, CilOCT’s unique use of a coordinate system with the scleral spur as the point of origin and the intersections between upper ciliary muscle border and perpendiculars to the sclera as basis for the abscissa renders the analysis independent of the ciliary muscle’s position and orientation within the OCT image [20]. CMT profiles of subjects with different refractive errors, of different ages, or in different accommodative states can thereby be accurately overlaid and compared. In addition to the commonly used ciliary muscle area measurements and discrete CMT readings, CMT profiles offer a novel analysis method for visualization and measurement of fine details of the thickness changes, which might remain undetected using current analysis techniques.

## conclusion-

after reading the study- I must admit that the format or order of the study made me confused- what was the propose of this study? if 3 previous studies were already puclished what is new in the current one- if it a review please descrivvve in depth the previous studies (n, methods etc) summeried the results etc.

The aim of the article is the introduction of CilOCT, a custom-developed open source software we applied successfully for segmenting the ciliary muscle in three of our previously published studies. The results of the three studies were summarized in the Discussion section but are now moved to the Results section. We think that sharing tools applied for analyzing research data is very important, especially in terms of reproducibility and comparability of outcomes. Therefore, we want to provide the research community with CilOCT to foster the research of the ciliary muscle morphology using OCT. To the best of our knowledge, this is the first software that is publicly available in this research area. Most other studies either used the in-built features of the OCT software (which usually does not provide distortion correction or precise measurement tools) or used self-developed proprietary software that is not available for other research groups.

In the revised Results section, we provide more information about our previous studies, but also refer to the respective articles for details.

I think if the aim is to present a new software the the format should be technical and present data that show that the software is more accurate them manul segmention that it reduce cost and time etc-

Unfortunately, it is not possible to provide the requested information, since we only have our data at our disposal and no means to compare it to the ones of others. It was not possible to obtain the software used by other groups to compare their analysis with ours. By offering our software as open-source to the research community, we aim to facilitate the comparison of the results derived from different approaches or software tools and thereby foster reproducibility of research.

# Reviewer #3

This manuscript summarizes the authors’ experience with a software for the analysis of OCT images of the ciliary muscle. This experience is based on 3 of the authors’ previous studies (ref 16, 18 and 19). The paper is well written, and demonstration of the technique is clear and appears to be easy to reproduce. However, in my opinion, the authors did not provide adequate justification to publish this manuscript, since there is no evidence of any new original research compared to the 3 previous reports. This manuscript may benefit from being presented as a review.

We thank the Reviewer for the valuable comments. Indeed, the aim of the manuscript is the introduction of the CilOCT software and its availability as open-source rather than the presentation of new research data. We believe that providing research software used to analyze data along with the data itself is important for reproducible research. In accordance with the Reviewer’s suggestion, we changed the title of the manuscript to better reflect its aims as follows:

***“Review of the application of the open-source software CilOCT for semi-automatic segmentation and analysis of the ciliary muscle in OCT images“***

Additionally, we changed the Abstract and Introduction to point out that this is not a research article but a review of the application of the CilOCT software.

At list one of the figures was published in one of the authors’ previous studies, though this was adapted with permission. This seems inappropriate to me.

We replaced the respective figure since the license of the figure of the original publication is not compatible with CC BY 4.0.
